# ORAL FRAILTY IS ASSOCIATED WITH MULTIFACETED FRAILTY IN ELDERLY OUTPATIENTS AT COMMUNITY DENTAL CLINICS

**DOI:** 10.1093/geroni/igz038.530

**Published:** 2019-11-08

**Authors:** Misa Nishimoto, Tomoki Tanaka, Yutaka Watanabe, Hirohiko Hirano, Takeshi Kikutani, Tetsuro Sato, Kazuko Nakajo, Katsuya Iijima

**Affiliations:** 1 Institute of Gerontology, The University of Tokyo, Tokyo, Japan; 2 Institute of Gerontology, The University of Tokyo, Japan, Tokyo, Japan; 3 Tokyo Metropolitan Institute of Gerontology, Tokyo, Japan; 4 Division of Clinical Oral Rehabilitation, Nippon Dental University Graduate School of Life Dentistry at Tokyo, Tokyo, Japan; 5 Kanagawa Dental Association, Kanagawa, Japan; 6 Kanagawa Prefectural Government, Japan, Kanagawa, Japan

## Abstract

Aim For achieving healthy aging, frailty prevention is essential. Because it is reported that accumulated declines in multiple oral functions (i.e. oral frailty) could lead to frailty progression, detailed countermeasures for oral frailty are currently required. However, dentists of community dental clinics don’t even know a prevalence of oral frailty among outpatients. Thus, we aimed to identify the prevalence of oral frailty and to examine the association with frailty in outpatients at community dental clinics. Methods The subjects were elderly outpatients at dental clinics in Kanagawa, Japan. Frailty was assessed using the Kihon checklists (KCL); those with ≥8 KCL score were classified as frailty. Furthermore, multiple functions (physical, nutrition, and oral) were assessed using subscale of the KCL. Oral frailty was defined as ≥3 deteriorations out of 5 oral status (remaining teeth, chewing ability, articulatory oral motor skill, subjective difficulties in eating and swallowing). Results Of 1,699 outpatients (mean age, 75 ± 6.3 years old; 40% men), 12% were frailty and 21% were oral frailty. When adjusted by confounding factors such as age and sex, those with oral frailty were associated with higher prevalence of frailty (OR, 3.25; 95%CI, 2.34-4.53), decreased physical and oral functions (OR, 1.53; 95%CI, 1.07-2.16: OR, 8.14; 95%CI, 6.05-10.95, respectively). Conclusions Oral frailty was associated with multi-faceted frailty in outpatients at community dental clinics. In addition to the importance of maintenance of whole oral functions including treating teeth, our findings suggest that it is also indispensable to consider the multi-faceted frailty for elderly patients.

